# *CNNM2*-Related Disorders: Phenotype and Its Severity Were Associated With the Mode of Inheritance

**DOI:** 10.3389/fped.2021.699568

**Published:** 2021-09-16

**Authors:** Han Zhang, Ye Wu, Yuwu Jiang

**Affiliations:** Department of Pediatrics, Peking University First Hospital, Beijing, China

**Keywords:** *CNNM2*, epilepsy, intellectual disability/developmental delay, hypomagnesemia, DUF21

## Abstract

*CNNM2* (Cystathionine-β-synthase-pair Domain Divalent Metal Cation Transport Mediator 2) pathogenic variants have been reported to cause hypomagnesemia, epilepsy, and intellectual disability/developmental delay (ID/DD). We identified two new cases with *CNNM2* novel *de novo* pathogenic variants, c.814T>C and c.976G>C. They both presented with infantile-onset epilepsy with DD and hypomagnesemia refractory to magnesium supplementation. To date, 21 cases with *CNNM2*-related disorders have been reported. We combined all 23 cases to analyze the features of *CNNM2*-related disorders. The phenotypes can be classified into three types: type 1, autosomal dominant (AD) inherited simple hypomagnesemia; type 2, AD inherited hypomagnesemia with epilepsy and ID/DD; and type 3, autosomal recessive (AR) inherited hypomagnesemia with epilepsy and ID/DD. All five type 1 cases had no epilepsy or ID/DD; they all had hypomagnesemia, and three of them presented with symptoms secondary to hypomagnesemia. Fifteen type 2 patients could have ID/DD and seizures, which can be controlled with antiseizure medications (ASMs); their variations clustered in the DUF21 domain of CNNM2. All three type 3 patients had seizures from 1 to 6 days after birth; the seizures were refractory, and 1/3 had status epilepticus; ID/DD in these AR-inherited cases was more severe than that of AD-inherited cases; they all had abnormalities of brain magnetic resonance imaging (MRI). Except for one patient whose serum magnesium was the lower limit of normal, others had definite hypomagnesemia. Hypomagnesemia could be improved after magnesium supplement but could not return to the normal level. Variations in the CBS2 domain may be related to lower serum magnesium. However, there was no significant difference in the level of serum magnesium among the patients with three different types of *CNNM2*-related disorders. The severity of different phenotypes was therefore not explained by decreased serum magnesium. We expanded the spectrum of *CNNM2* variants and classified the phenotypes of *CNNM2*-related disorders into three types. We found that DUF21 domain variations were most associated with *CNNM2*-related central nervous system phenotypes, whereas hypomagnesemia was more pronounced in patients with CBS2 domain variations, and AR-inherited *CNNM2*-related disorders had the most severe phenotype. These results provide important clues for further functional studies of *CNNM2* and provide basic foundations for more accurate genetic counseling.

## Introduction

Magnesium is an important cation in the human body. It is involved in the function of a variety of enzymes in cells and associated with the excitability of nerve cells and muscles. Magnesium transporter-associated genes include *MMGT1, MAGT1, NIPAL1, NIPAL4, MRS2, CNNM2*, and *CNNM4*. *CNNM2*, previously known as *ACDP2* (Ancient Conserved Domain Protein 2), has been demonstrated to be related to magnesium homeostasis in humans (1). In 2011, Stuiver et al. reported that *CNNM2* pathogenic variants could cause hereditary hypomagnesemia (2). Subsequently, epilepsy and intellectual disability/developmental delay (ID/DD) had been added to the spectrum of phenotypes of *CNNM2* variants (3). Up to now, a total of five articles have reported 21 cases of *CNNM2*-related disorders (2–6). But the relationship between genotypes and phenotypes of *CNNM2*-related disorders remains unknown. In this study, we reported two new Chinese cases of hypomagnesemia with epilepsy and DD caused by novel identified *de novo* heterozygous variants. We also investigated the relationship between the phenotypes and the variant sites/mode of inheritance in all 23 cases related to *CNNM2* mutations including two cases we found and 21 cases previously reported.

## Materials and Methods

### Participants

Two Chinese cases with infantile-onset epilepsy and global DD had been identified to have novel *CNNM2* pathogenic variants. These two cases and previously reported 21 *CNNM2* mutation-related cases were collected for all available data, including clinical manifestations, electroencephalogram (EEG), brain MRI, serum electrolytes, gene variants information, and mode of inheritance.

### Variation Analysis

Written informed consent was obtained from the parents of both patients. This study was approved by the institutional review boards of Peking University First Hospital. We collected 5 ml of peripheral venous blood from patients and their parents. Genomic DNA was extracted for trio-whole exome sequencing (WES). The pathogenicity of the variants was predicted by SIFT, PolyPhen-2, CADD, and MutationTaster. Variants were evaluated against the overall population in 1000 Genomes, gnomAD, and ExAC databases. The pathogenicity was classified according to variant classification standards based on the American College of Medical Genetics and Genomics (ACMG) guidelines, 2015.

## Results

### Clinical Features

#### Case 1

Case 1 was a 2-year-old boy who carried a novel identified *de novo* missense heterozygous variant c.814T>C [p.Phe272Leu]. He still could not raise his head steadily at 6 months of age. His seizures started at 10 months after birth, mainly characterized by eyes gazing to the left side and perioral cyanosis, sometimes accompanied by jaw tremor, right lower limb raising, and hand tremor. EEG showed focal frontal epileptic discharge. Although ictal EEG of this patient was not available, the electrical-clinical features indicate that his seizures were focal seizures. The seizures were clustered, up to eight attacks per day, only 2–3 days per month. He had been seizure-free from 12 months of age by levetiracetam monotherapy. He had DD. When he was 1 year old, he could sit and crawl without others' help. He could walk without support from 1 year 3 months of age, but he still could not walk steadily now. He began to vocalize at 1 year old, but he could not make the sound of “babamama” till now. He had no apparent malformations. Brain MRI was normal. His initial serum magnesium was 0.56 mmol/L. After his seizures were completely controlled, he was diagnosed with causative gene and began to receive magnesium supplement at 1 year old. But diarrhea occurred after oral administration of magnesium, which was improved after suspension of magnesium supplementation. It was considered that diarrhea was related to gastrointestinal side effects of oral magnesium, so the treatment with magnesium supplementation was interrupted, and his parents did not monitor serum magnesium level for him regularly. During magnesium supplementation treatment, his highest serum magnesium was 0.62 mmol/L and he had no seizures, but no improvement in psychomotor development was observed.

#### Case 2

**Figure 1:**
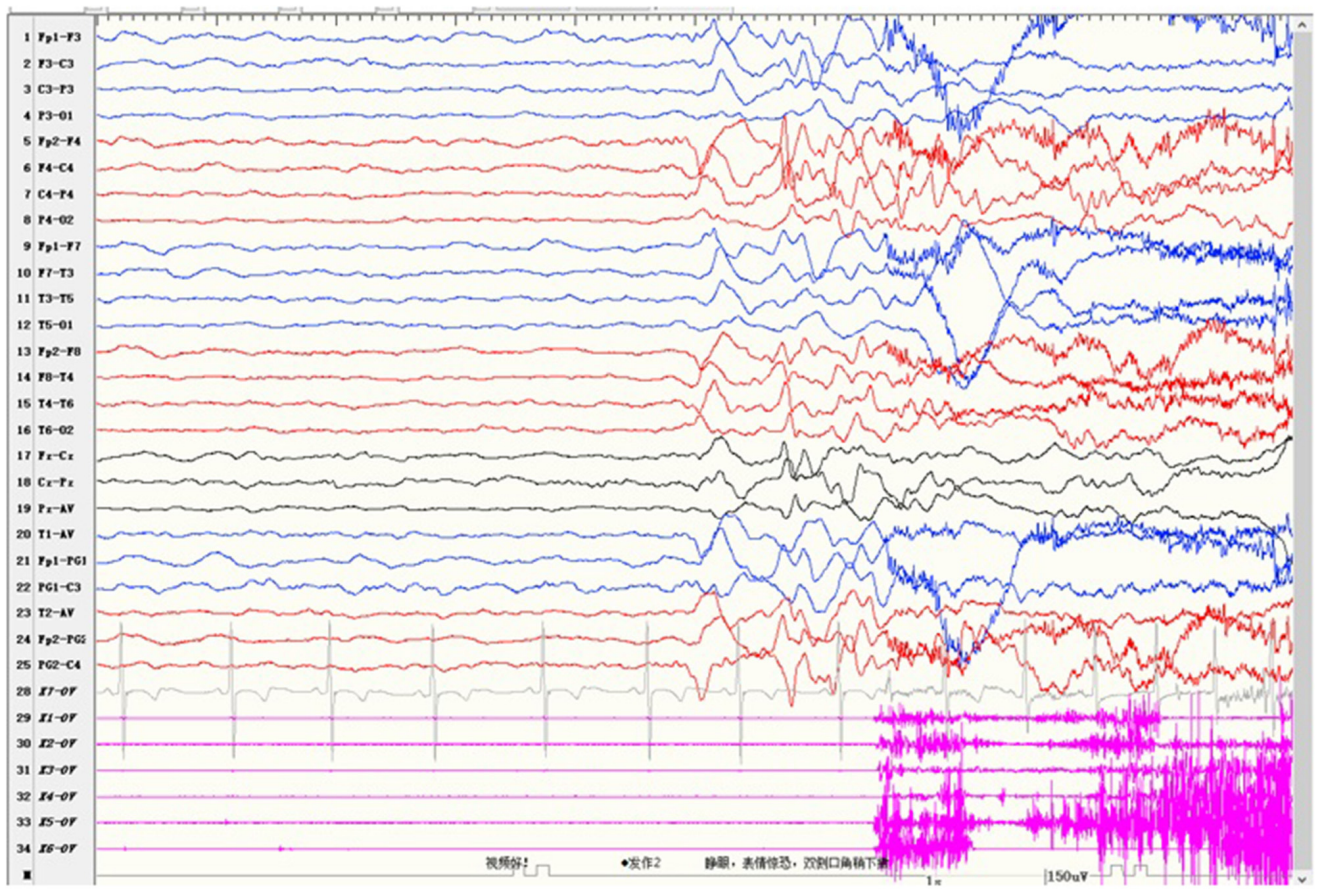
Ictal electroencephalogram of case 2 showed the seizure onset from the right hemisphere.

Case 2 was a 4-year-old girl who carried a novel identified *de novo* missense heterozygous variant c.976G>C [p.Val326Leu]. Her seizures started at 8 months after birth and always occurred during sleep. Seizures involved eye opening and fearful expression, eye and head turning left, and then tonic and clonic jerks of the four limbs. Seizures were accompanied by perioral cyanosis. Ictal EEG showed the seizure onset from the right hemisphere (Figure 1). She was treated with sodium valproate at first, and the frequency of seizures decreased. Levetiracetam and lamotrigine adjunctive therapy had not shown any effect. Treatment with oxcarbazepine was discontinued due to allergic rash. Lacosamide was started 1 month before the last follow-up. Now she is treated with sodium valproate and lacosamide, and no seizure was seen in the recent 1 month. Gesell's developmental scale revealed severe DD. When she was 4 years old, she could recognize animal pictures, but she could not speak their names. Now she still cannot say anything but “baba” and “mama”. Physical examination revealed no apparent malformations. Brain MRI was normal. Her initial serum magnesium was 0.44 mmol/L. She tolerates oral magnesium supplementation at a dose of 0.1–0.2 mmol/kg/day, and her serum magnesium elevated to 0.52 mmol/L. But her serum magnesium could not return to the normal level.

### Whole Exome Sequencing Results

Both cases were identified *de novo* heterozygous variants in *CNNM2* by trio-WES, which were c.814T>C [p.Phe272Leu] and c.976G>C [p.Val326Leu]. We used multiple *in silico* tools to analyze these variants' conservation, pathogenicity, and minor allele frequency (MAF). Finally, according to the ACMG guidelines, both variants were clarified as likely pathogenic (LP) (Table 1). Other pathogenic variants associated with hypomagnesemia, epilepsy, and ID/DD were not found by trio-WES.

**Table 1:** Analysis of pathogenicity of the variants in *CNNM2*.

**Case**	**Gene**	**Variant**	**Variant origin**	**MAF**			**PolyPhen-2**	**SIFT**	**Mutation Taster**	**CADD**	**Evidence**	**Category**
				**1000 Genomes**	**ExAC**	**gnomAD**						
1	*CNNM2*	c.814T>C [p.Phe272Leu]	*De novo*	NE	NE	NE	0.81	0.13	DC	25.3	PS2+PM2+PP3	LP
2	*CNNM2*	c.976G>C [p.Val326Leu]	*De novo*	NE	NE	NE	0.18	0.01	DC	24.2	PS2+PM2+PP3	LP

*Transcript: NM_017649. NE, not existing; DC, disease causing; LP, likely pathogenic*.

### Phenotype and Genotype Relation Analysis

**Figure 2:**
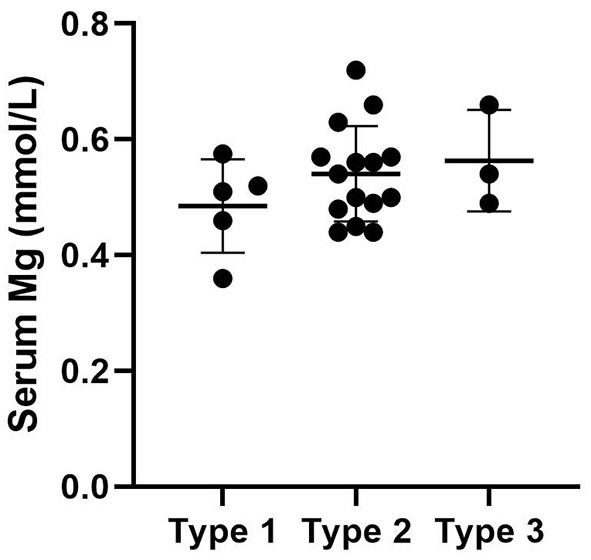
Initial serum magnesium in patients with three types of *CNNM2*-related disorders. No significant difference was found. *p* = 0.3496.

**Figure 3:**
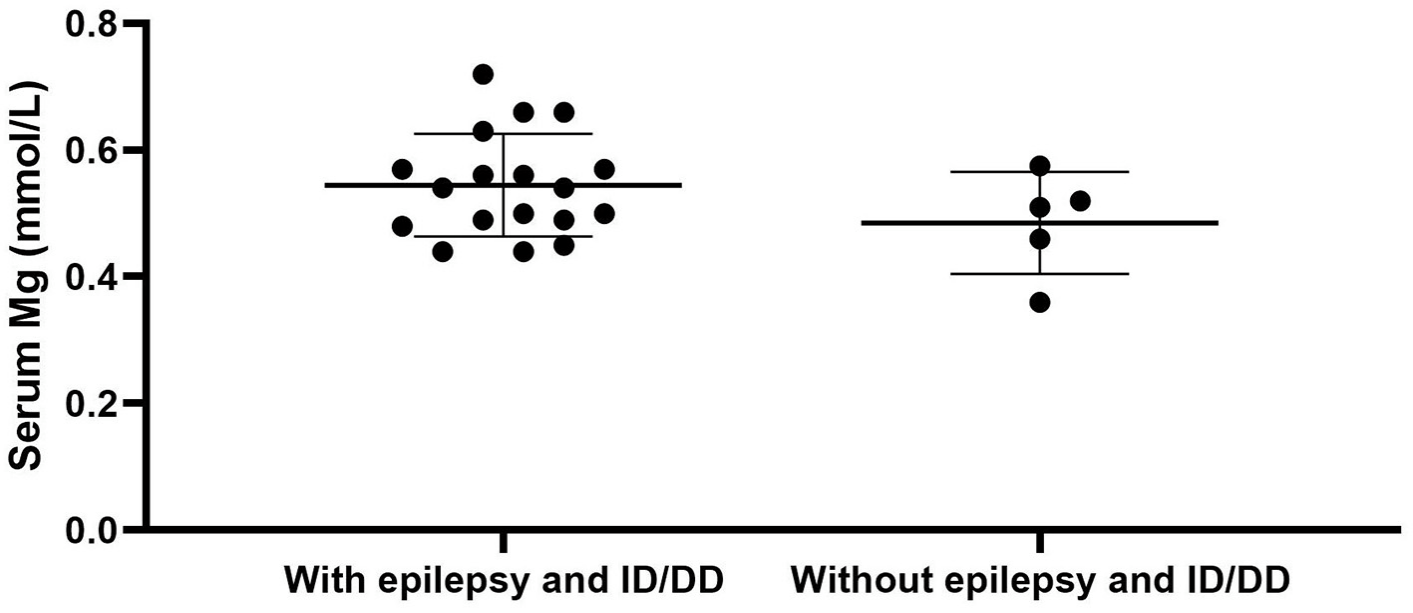
Initial serum magnesium in patients with epilepsy and ID/DD (type 2 and type 3) and without epilepsy and ID/DD (type 1). No significant difference was found. *p* = 0.3905. ID/DD, intellectual disability/developmental delay.

We used “CNNM2” as the keyword to search on PubMed. Excluding variants of unknown significance or non-pathogenic, there are totally 23 cases with CNNM2-related disorders, including the two cases we reported here (2–6) (Table 2). The features of these cases can be classified into three types: type 1, autosomal dominant (AD)-inherited simple hypomagnesemia; type 2, AD-inherited hypomagnesemia with epilepsy and ID/DD; and type 3, autosomal recessive (AR)-inherited hypomagnesemia with epilepsy and ID/DD. Type 1, AD-inherited simple hypomagnesemia, was the first phenotype to be discovered. The onset of hypomagnesemia was insidious. The age at which hypomagnesemia was found varied from 1 to 16 years. Two of five cases of type 1 were asymptomatic, and their hypomagnesemia was detected in serum electrolyte tests; the other cases had atypical clinical symptoms associated with hypomagnesemia, including muscle spasms, headache, fatigue, and vertigo. Type 1 patients had no epilepsy or ID/DD. Their initial serum magnesium was 0.36–0.575 mmol/L; it was reported that the serum magnesium could be increased to 0.61–0.64 mmol/L in two cases by magnesium supplementation but could not return to the normal level. Type 2, AD-inherited hypomagnesemia with epilepsy and ID/DD, is the most common phenotype in cases with *CNNM2* variant (15/23). Most of the cases are with seizures at onset. In type 2 cases, 86.7% (13/15) had epileptic seizures, and 93.3% (14/15) had ID/DD in the course of the disease. Based on limited available details, the age at seizure onset was 4 months to 1 year in most cases, but 16 years of age in one case. Their seizures showed good response to antiseizure medications (ASMs); multiple kinds of ASMs had been reported to be effective, including phenobarbital, valproic acid, clobazam, levetiracetam, and lacosamide. All type 2 patients had ID/DD, characterized by language expression dysfunction. Physical development was normal in type 2 cases. Physical examination revealed no apparent malformations. Brain MRI was normal except for corona radiata and centrum semiovale white matter T2 slightly hyperintense in one case. Of the cases, 93.3% had definite hypomagnesemia; their initial serum magnesium was 0.44–0.66 mmol/L. However, borderline hypomagnesemia was found in one case whose initial serum magnesium was 0.72 mmol/L. Hypomagnesemia could be improved after magnesium supplement but could not return to the normal level. Type 3, AR-inherited hypomagnesemia with epilepsy and ID/DD, was relatively rare. Only three cases had been reported. But this is the most severe type of *CNNM2-*related disorders. Their seizures onset from 1 to 6 days after birth, much earlier than that of type 2. They could have myoclonic seizures, generalized tonic–clonic seizures, and status epilepticus. In type 3 cases, multiple ASMs were used, but seizures were refractory; valproic acid, levetiracetam, lamotrigine, and topiramate may decrease the frequency of seizures. All type 3 cases had severe ID/DD, and language development was most severely delayed. They all had brain MRI abnormalities, including dysmyelination and progressive cerebral cortical atrophy. The patient who carried c.1642G>A homozygous variant had more severe clinical manifestations, including swallowing difficulties, recurrent aspiration pneumonias, bilateral optic disc pallor, bone metabolism disorder, and facial abnormalities, which were not seen in the two patients who carried c.364G>A homozygous variants. In contrast, hypomagnesemia was no more severe in this severe type than in the other two types. Initial serum magnesium was 0.38–0.5 mmol/L. Serum magnesium could be increased to 0.49–0.66 mmol/L after magnesium supplement but could not be corrected normal. Table 3 demonstrates the features of these three types of *CNNM2*-related disorders. We used one-way ANOVA to compare the initial serum magnesium in patients with three types of *CNNM2*-related disorders, and no significant difference was found (Figure 2). And we used the Mann–Whitney test to compare the initial serum magnesium in patients with epilepsy and ID/DD (type 2 and type 3) and without epilepsy and ID/DD (type 1); no significant difference was found either (Figure 3).

**Table 2:** The variants and clinical phenotypes of patients with *CNNM2*-related disorders.

**Case**	**Variant**	**Amino acid changes**	**Homo/ het**	**Mode of inheritance**	**Gender**	**Epilepsy**				**ID/DD**	**Hypomagnesemia**			**Physical development**			**Others**
						**With/without**	**Onset age**	**Refractory seizures**	**Effective ASM**		**Initial serum Mg (mmol/L)**	**Serum Mg after supplementation (mmol/L)**	**Symptoms**	**Microcephaly**	**Structural abnormalities of brain**	**Malnutrition**	
1 (2)	c.117delG	p.Ile40SerfsX15	Het	AD	M	–				–	0.46	NA	Muscle spasms, headache	–	–	–	
2 (2)	c.117delG	p.Ile40SerfsX15	Het	AD	F	–				–	0.51	0.64	Muscle spasms, stuttering LOC	–	–	–	
3 (2)	c.1703C>T	p.Thr568Ile	Het	AD	F	–				–	0.52	NA	-	–	–	–	
4 (2)	c.1703C>T	p.Thr568Ile	Het	AD	M	–				–	0.36	0.61	Weakness, vertigo, headache	–	–	–	
5 (3)	c.364G>A	p.Glu122Lys	Homo	AR	M	+	1 day	+	VPA, LTG	Severe	0.5	0.66	–	+	Myelination defects, opercularization defect, widened outer cerebrospinal liquor spaces, calcification of basal ganglia	–	
6 (3)	c.364G>A	p.Glu122Lys	Homo	AR	F	+	6 days	+	VPA, LEV	Severe	0.5	0.54	–	+	Calcification of basal ganglia	–	
7 (3)	c.1069G>A	p.Glu357Lys	Het	AD	F	+	7 months	–	PB	Moderate	0.56	0.56	–	NA	–	–	
8 (3)	c.806C>G	p.Ser269Trp	Het	AD	F	+	1 year	–	VPA	Moderate	0.44	0.53	–	NA	–	–	
9 (3)	c.1069G>A	p.Glu357Lys	Het	AD	M	+	4 months	–	CLB	Moderate	0.5	0.68	–	NA	–	–	
10 (3)	c.988C>T	p.Leu330Phe	Het	AD	F	+	16 years	–	Unknown	Mild	0.66	Unknown	–	NA	–	–	
11 (4)	c.1642G>A	p.Val548Met	Homo	AR	M	+	1 day	+	TPM	Severe	0.38	0.49	-	NA	Cerebral cortical atrophy, global reduction of white matter	Normal at birth; height, weight and head circumference all less than P3 at 15 years	Hypotonia, swallowing difficulties, recurrent aspiration pneumonias; bilateral optic disc pallor; abnormal bone metabolism; facial abnormalities
12 (5)	c.2384C>A	p.Ser795*	Het	AD	M	–				–	0.575	NA	–	NA	NA	NA	
13 (7)	g.(?_104678237) _(104816721_?) del		Het	AD	F	+	NA	NA	NA	+	0.63	0.65	NA		–	NA	
14 (7)	g.104814162_ 104814164 del		Het	AD	M	+	NA	NA	NA	+	0.57	NA	NA	NA	–	NA	
15 (7)	c.143T>C	p.Leu48Pro	Het	AD	M	+	NA	NA	NA	–	0.45	0.53–0.66	NA	NA	–	NA	
16 (7)	c.942C>G	p.Tyr314*	Het	AD	M	–				+	0.48	NA	NA	NA	–	NA	
17 (7)	c.961_963del	p.Leu321del	Het	AD	M	+	NA	NA	NA	+	0.5	0.51	NA	NA	–	NA	
18 (7)	c.970G>A	p.Val324Met	Het	AD	M	+	NA	NA	NA	+	0.54	0.52	NA	NA	–	NA	
19 (7)	c.1253T>C	p.Leu418Pro	Het	AD	F	+	NA	NA	NA	+	0.49	0.58	NA	NA	–	NA	
20 (7)	c.2384C>T	p.Ser795Leu	Het	AD	F	–				+	0.72	0.7	NA	NA	–	NA	
21 (7)	c.2389C>T	p.Arg797*	Het	AD	F	+	NA	NA	NA	+	0.57	0.69	NA	NA	Corona radiata and centrum semiovale white matter T2 slightly hyperintense	NA	
22	c.814T>C	p.Phe272Leu	Het	AD	M	+	6 months	–	LEV	+	0.56	/	–	–	–	–	
23	c.976G>C	p.Val326Leu	Het	AD	F	+	8 months	Unknown	VPA, LCM?	Severe	0.44	0.52	–	–	–	–	

*Case 1 is the father of case 2. Case 3 is the mother of case 4. Case 5 and case 6 were the two siblings of same suspected consanguineous parents. Case 11 was the sibling of consanguineous parents. Case 12 had two family members carrying the same variant, both with insidious hypomagnesemia. Case 15 had six family members carrying the same variant, they all presented with hypomagnesemia, but only three of them had defects in motor skills or speech, and none of them had seizures. ID/DD, intellectual disability/developmental delay; ASM, antiseizure medication; AD, autosomal dominant; AR, autosomal recessive; VPA, valproic acid; LEV, levetiracetam; LOC, locus of control; LTG, lamotrigine; PB, phenobarbital; CLB, clobazam; TPM, topiramate; LCM, lacosamide*.

**Table 3:** Three types of *CNNM2*-related disorders and their features.

**Type**	**1**	**2**	**3**
Phenotype	AD-inherited simple hypomagnesemia	AD-inherited hypomagnesemia with epilepsy and ID/DD	AR-inherited hypomagnesemia with epilepsy and ID/DD
Number of cases	5/23	15/23	3/23
Age of onset	1–16 years	5/6 4 months−1 year, 1/6 16 years	1–6 days
Epilepsy	-	13/15 Focal seizures, easy to control with ASMs	3/3 Multiple forms, refractory seizures, may have status epilepticus
Psychomotor development	Normal	14/15 Mild to severe ID/DD, language expression inability	3/3 Severe ID/DD, nonverbal
Apparent malformations	–	–	May have facial abnormalities
Brain MRI abnormalities	–	–	+
Hypomagnesemia	+	+	+
Initial serum magnesium (mmol/L)	0.36–0.575	0.44–0.72	0.38–0.5
Others			Swallowing difficulties, recurrent aspiration pneumonias; bilateral optic disc pallor; abnormal bone metabolism
Mode of inheritance	AD	AD	AR

*AD, autosomal dominant; ID/DD, intellectual disability/developmental delay; AR, autosomal recessive; ASM, antiseizure medication*.

**Figure 4:**
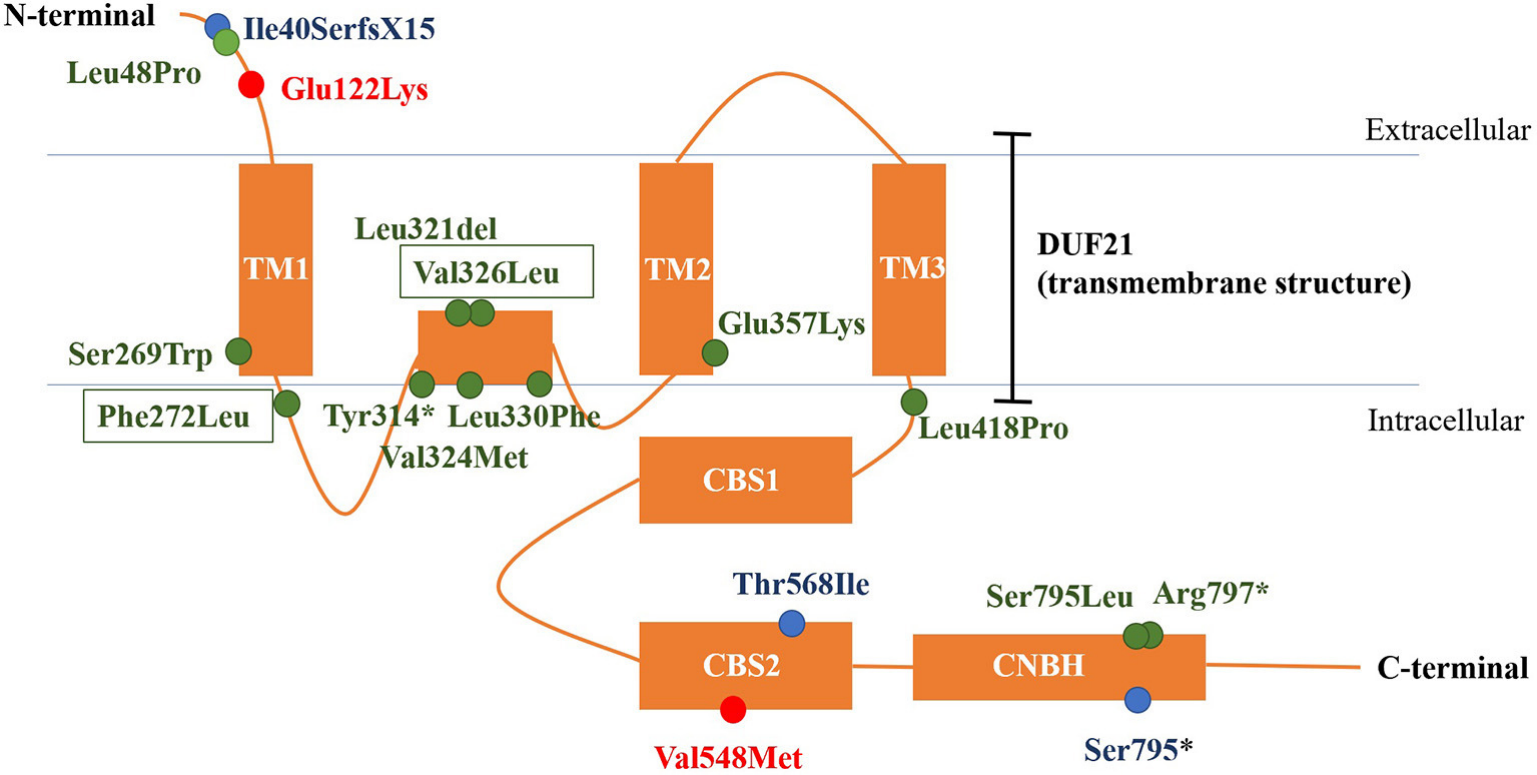
The structure of CNNM2 and the sites of variations. Blue, AD-inherited simple hypomagnesemia; red, AR-inherited hypomagnesemia with epilepsy and ID/DD; green: AD-inherited hypomagnesemia with epilepsy and ID/DD; black box, our cases. AD, autosomal dominant; AR, autosomal recessive; ID/DD, intellectual disability/developmental delay.

**Figure 5:**
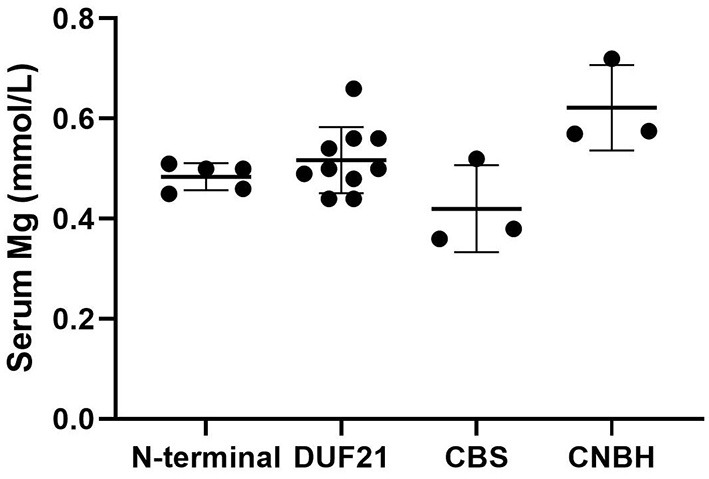
Initial serum magnesium of patients with variants of different domains. We used one-way ANOVA to compare the difference. *p* = 0.20.

*CNNM2* is located on chromosome 10q24.32. CNNM2 protein is a transmembrane protein on the cell membrane and is composed of 875 amino acids. CNNM2 contains one DUF21 domain, two CBS domains, and one CNBH (Cyclic Nucleotide-Binding Homology) domain (8). Variants caused *CNNM2*-related disorders type 1 were frameshift variant, missense variant, and stop-gain variant. The changed amino acids were located on various domains and include extracellular segment near the N-terminal, the second CBS domain, and the CNBH domain, but were not within the DUF21 domain (Figure 4). Variants caused *CNNM2*-related disorders type 2 included missense variants, stop-gain variants, deletion variant, and two deletion copy number variants (CNVs). Of the variant site, 75% is located in or immediately adjacent to the DUF21 domain. All variants that caused *CNNM2*-related disorders type 3 were missense variants. One variant was located on the extracellular segment near the N-terminal, and another was located on the second CBS domain, whereas the latter variants cause a more severe phenotype. It was noteworthy that all variants located in DUF21 domain caused type 2 of *CNNM2*-related disorders. Among all 23 cases, patients with variants on CBS domain had lower serum magnesium, but the difference was not significant (Figure 5).

## Discussion

Magnesium plays an important role in the human body. It is the fourth most abundant cation and is a constituent of a variety of enzymes in the body that are involved in various important metabolic processes including DNA synthesis, protein synthesis, and oxidative phosphorylation (9). Besides, magnesium ions (Mg^2+^) have an inhibitory effect on the excitability of nerve cells in the central nervous system, skeletal muscle, and myocardium. Normal serum magnesium is 0.7–1.1 mmol/L. Neuromuscular irritability, tremor, hypokalemia, and hypocalcemia present when serum magnesium is less than 0.7 mmol/L. If serum magnesium is less than 0.4 mmol/L, hypomagnesemia can cause more severe clinical symptoms, including tetany, nystagmus, seizures, mental disorders, and arrhythmias (10). *CNNM2* has been demonstrated to play a role in magnesium homeostasis in humans (1).

We summarized and clarified phenotypes of *CNNM2*-related disorders into three types: AD-inherited simple hypomagnesemia; AD-inherited hypomagnesemia with epilepsy and ID/DD; and AR-inherited hypomagnesemia with epilepsy and ID/DD. Type 1, AD-inherited simple hypomagnesemia, has an insidious onset without epilepsy and ID/DD. Some type 1 cases had symptoms associated with hypomagnesemia, including muscle spasms, headache, fatigue, and vertigo; however, the others were asymptomatic. Type 2, AD-inherited hypomagnesemia with epilepsy and ID/DD, mostly presented with ASM-effective seizures at onset at the age of 4 months to 1 year, and ID/DD is characterized by language expression inability. Type 3, AR-inherited hypomagnesemia with epilepsy and ID/DD, presented with more severe phenotypes. Multiple forms of seizures present at 1–6 days after birth, including status epilepticus. The seizures were refractory. ID/DD of type 3 is more severe than type 2. Patients with type 3 could have brain and facial abnormalities. Therefore, *CNNM2*-related disorders should be considered if a patient presented with any one of the three phenotypes.

All patients who carried pathogenic variants in *CNNM2* had hypomagnesemia. Some of them were asymptomatic. However, serum magnesium could not be corrected to the normal level by magnesium supplement. There was no significant difference in serum magnesium levels between different types of cases. Hypomagnesemia could not be correlated to seizures directly in these cases, because the seizures could not be controlled by magnesium supplement. Differences in the severity could not be explained by differences in the degree of decreased serum magnesium. The phenotype of AR-inherited hypomagnesemia with epilepsy and ID/DD was more severe than that of AD-inherited, which suggests that the causative variant of *CNNM2* may be loss of function (LOF). Some pathogenic missense variants have been proved as LOF variants (6). However, this conclusion still needs further functional studies to be confirmed. In AD-inherited *CNNM2*-related disorders, the basis of abnormal protein function caused by pathogenic variants may be haploinsufficiency.

*CNNM2* is highly expressed in the brain and kidney, while it is widely expressed in various organs including the digestive tract, cardiovascular system, lungs, endocrine glands, and blood cells (11). *CNNM2* variants may lead to serum magnesium reduction by attenuating the reabsorption of magnesium at the distal convoluted tubule in the kidney (2). However, the exact function of CNNM2 protein and its mechanism are still unknown. CNNM2 contains one DUF21 domain, two CBS domains, and one CNBH domain (1). The DUF21 domain contains three transmembrane structures and one intramembrane structure, and the specific function is currently unknown. However, this domain is present in all proteins of the CNNM family and is highly conserved from prokaryotic to eukaryotic cells, suggesting that it may have an important biological function. The two CBS domains (also known as Bateman modules) are demonstrated to be closely related to the function of the protein (12). They may dimerize by binding with Mg^2+^-ATP and alter the conformation of the CNNM2 protein (8). Inactivation of the CNBH domain can cause loss of CNNM2 function (13); however, the function of this domain is unknown. It is thought that its dimerization can assist the CBS domain to function. The mechanisms by which CNNM2 regulates magnesium homeostasis are still under discussion.

Most investigators suggest that CNNM2 itself is a transporter for Mg^2+^ (8). Immunohistochemical staining of human kidney sections confirmed that *CNNM2* is expressed in the distal convoluted tubule, which is the last site of Mg^2+^ reabsorption (2). Low magnesium caused high expression of *Cnnm2* on the lateral side of the basement membrane of the distal convoluted tubule in the rat kidney, suggesting that CNNM2 may be related to the transport of magnesium from within renal tubular epithelial cells into capillaries. Thereafter, in HEK293 cell line-based experiments, it was found that the influx of sodium ions was decreased in cells transfected with the *CNNM2* p.Thr568Ile mutation (a mutation causing hereditary hypomagnesemia) compared with wild type. Therefore, it is thought that CNNM2 protein is involved in the reverse transport of magnesium and sodium ions in the distal convoluted tubule of the kidney, transporting magnesium ions from within renal tubular epithelial cells to capillaries (14). Besides, Tremblay's (15) and Miki's (16) team found that the interaction between CNNMs and PRL (phosphatases of the regenerating liver) was associated with tumorigenesis in 2014. PRL is a molecule highly expressed in solid and hematologic tumor cells (17, 18). PRLs can form complexes with CNNMs, thereby inhibiting the activity of CNNM to transport Mg^2+^ extracellularly, increasing the intracellular concentration of Mg^2+^, and promoting the growth of tumor cells. In breast cancer cells, when the intracellular Mg^2+^ concentration is low, the expression of PRL-1 can be increased. PRL-1 can anchor the CBS domain, after which the charge interaction between two adjacent proteins alters the conformation of the CBS domain, which causes changes in the structure of the transmembrane region of the protein, allowing magnesium ion influx. Also, Mg^2+^-ATP can bind to the CBS domain as the intracellular Mg^2+^ concentration increases, to maintain the state of transporter opening. However, this mechanism cannot explain the mechanism of CNNM2 in the distal convoluted tubule of the kidney. And the function and role of CNNM2 in the central nervous system are still unknown. Some investigators have also suggested that CNNM2 is not a Mg^2+^ transporter *per se* but a factor affecting Mg^2+^ transporters (7).

Different phenotypes of *CNNM2*-related disorders are associated with different modes of inheritance and different domains in which variation occurs. Variation of AD-inherited simple hypomagnesemia (type 1) contains a frameshift variant located near the N-terminal (p.Ile40SerfsX15), a missense variant in the CBS domain (p.Thr568Ile), and a stop-gain variant located in the CNBH domain, where p.Ile40SerfsX15 and p.Ser795^*^ can cause changes in mRNA and protein length, and the structurally abnormal mRNA and proteins may be degraded, causing a decrease in intracellular *CNNM2* expression, which affects the transmembrane transport of magnesium. p.Thr568Ile is located in the CBS2 domain in the core region of the CNNM2, which is highly conserved, and the variation may cause a loss in CNNM2 function. Variation of AD-inherited hypomagnesemia with epilepsy and ID/DD (type 2) included missense variants, stop-gain variants, deletion variant, and two heterozygous deletion CNVs. Most of them (p.Ser269Trp, p.Phe272 Leu, p.Tyr314^*^, p.Leu321del, p.Val324Met, p.Val326Leu, p.Leu330Phe, p.Glu357Lys, and p.Leu418Pro) clustered in the DUF21 domain, the transmembrane structure of CNNM2 with unknown function. And five of the nine variant sites are located in the intramembrane structure. These patients had manifestations of central nervous system involvement independent of hypomagnesemia, including ID/DD and epilepsy, suggesting that functional abnormalities in DUF21 domain may be more closely related to neurological function. The pathogenic mechanism may be abnormal transmembrane structure caused by mutations, rather than a decreased expression of *CNNM2*, causing abnormal neuron excitability. This suggests that in addition to affecting magnesium reabsorption in the kidney, the function of CNNM2 may also include participating in the regulation of neural cell excitability in the central nervous system, and the important core of regulatory function may be the transmembrane region DUF21. p.Leu48Pro is located near the N-terminal, and this site was located in a region that crosses the endoplasmic reticulum (ER) membrane during protein transport (6). This suggested that p.Leu48Pro may affect the transport of CNNM2 protein to the cell membrane, which in turn reduced the amount of CNNM2 protein on the cell membrane. However, p.Ser795Leu and p.Arg 797^*^ are located in the CNBH domain, and another pathogenic variant at Ser795 (p.Ser795^*^) was related to type 1 *CNNM2*-related disorders. This may suggest that the region near Ser795 has a relatively important effect on the function of the CNBH domain. But the pathogenic mechanisms of these three variants could not yet be well-explained. Variations of AR-inherited hypomagnesemia with epilepsy and ID/DD (type 3) are missense variants, including p.Glu122Lys located between the N-terminal and DUF21 domain and p.Val548Met located in CBS domain. p.Glu122Lys is adjacent to the DUF21 domain, and this variant causes a change in the charge of the amino acid residue, suggesting that some regions of the N-terminal of CNNM2 may form some interaction with the DUF21 domain electrostatically, which, like the variants of type 2, alters the membrane structure of DUF21 domain, causing changes in neuron membrane excitability, resulting in similar central nervous system manifestations. p.Val548Met is located in the CBS domain of CNNM2, and this variant causes the most significant decrease in serum magnesium and the most severe neurological phenotype, suggesting that this variant site may be the core position of the CBS domain, and the amino acid changes caused by this variant significantly affect the function of the CNNM2. The patient carried p.Val548Met had initial serum magnesium of 0.38 mmol/L; however, considering that p.Thr568Ile carriers had equally severe hypomagnesemia but did not develop any central nervous system phenotype, this indicated that the remarkable hypomagnesemia *per se* cannot explain the severe phenotype of the central nervous system in this patient. Therefore, the mechanism by which p.Val548Met leads to severe CNS phenotypes may be at least partially related to the DUF21 domain, as in type 2 cases.

Although all patients with *CNNM2*-related disorders had hypomagnesemia, the serum magnesium was different in each case, which may be related to the protein domain where the variant was located. Among the 23 reported cases, those with variants in the CBS domain had lower serum magnesium levels, and the lowest two had variants both located in the CBS2 domain (p.Val548Met and p.Thr568Ile). This suggests an important role for the CBS domain in the Mg^2+^ transport function of CNNM2.

In summary, we reported two cases first in the Chinese population with hypomagnesemia, epilepsy, and DD caused by novel *de novo* heterozygous variants in *CNNM2* (c.814T>C [p.Phe272Leu] and c.976G>C [p.Val326Leu]). We summarized and classified the phenotypes of *CNNM2*-related disorders into three types. We found that *CNNM2* related central nervous system phenotypes were most associated with DUF21 domain variations, whereas hypomagnesemia was more pronounced in patients with CBS2 domain variations, and AR-inherited *CNNM2*-related disorders had the most severe phenotype. The limitation of our study is the relatively small sample size. The features summarized from 23 patients might not be the real characteristics for such a complicated disease. More cases and further biological functional studies are needed to confirm, modify, and interpret our findings. However, our findings provide important clues for mechanism studies of *CNNM2*-related disorder and provide the possibility for accurate genetic counseling.

## Data Availability Statement

The original contributions presented in the study are included in the article/supplementary material, further inquiries can be directed to the corresponding author/s.

## Ethics Statement

Written informed consent was obtained from the minor(s)' legal guardian/next of kin for the publication of any potentially identifiable images or data included in this article.

## Author Contributions

YJ: study design and revision of the manuscript. YW and YJ: collection of clinical and WES data. HZ: follow up the patients' information and analyses and draft preparation. All authors contributed to the article and approved the submitted version.

## Funding

This work was supported by the National Key Research and Development Program of China (grant numbers: 2020YFA0804003, 2016YFC1306201, and 2016YFC0901505), by the National Natural Science Foundation of China (grant numbers: 81971211, 12026606, and 81601131), by Beijing Natural Science Foundation (grant number: 7212109), by the Beijing Key Laboratory of Molecular Diagnosis and Study on Pediatric Genetic Diseases (grant number: BZ0317), and by the Fundamental Research Funds for the Central Universities (grant numbers: BMU2017JI002, BMU2018XY006, and PKU2017LCX06). The authors declare no competing financial interests. The funding agencies had no role in the study design, the experiments, analysis, or interpretation of data, the writing of the report, or the decision to submit the article for publication.

## Conflict of Interest

The authors declare that the research was conducted in the absence of any commercial or financial relationships that could be construed as a potential conflict of interest.

## Publisher's Note

All claims expressed in this article are solely those of the authors and do not necessarily represent those of their affiliated organizations, or those of the publisher, the editors and the reviewers. Any product that may be evaluated in this article, or claim that may be made by its manufacturer, is not guaranteed or endorsed by the publisher.
